# Gender Differences in Anxiety, Depression, and Fertility-Related Quality of Life Among Infertile Couples: A Cross-Sectional Study

**DOI:** 10.3390/jcm15166271

**Published:** 2026-08-13

**Authors:** Hatice Yilmaz Dogru, Cuma Tasin

**Affiliations:** 1 Department of Obstetrics and Gynecology, Mersin City Training and Research Hospital, 33240 Mersin, Turkey; 2 Department of Perinatology, Mersin City Training and Research Hospital, 33240 Mersin, Turkey; cumatasin@gmail.com

**Keywords:** infertility, anxiety, depression, quality of life, FertiQoL, psychological distress, gender differences

## Abstract

**Background/Objectives**: Infertility is associated with considerable psychological distress and impaired quality of life; however, gender-related differences in these outcomes remain unclear. This study aimed to compare anxiety, depression, and fertility-related quality of life between women and men in infertile couples and to examine the relationships between psychological distress and fertility-specific quality of life. **Methods**: This cross-sectional study included 75 infertile women and their male partners attending the Reproductive Health and Infertility Outpatient Clinic. Anxiety and depressive symptoms were assessed using the Beck Anxiety Inventory (BAI) and Beck Depression Inventory-II (BDI-II), respectively. Fertility-related quality of life was evaluated using the Fertility Quality of Life (FertiQoL) questionnaire. Group comparisons and correlation analyses were performed using appropriate statistical tests. **Results**: Females had significantly higher total anxiety scores than males (10.44 ± 10.04 vs. 6.18 ± 7.86, *p* = 0.004). Females also showed higher affective depressive symptom scores (3.21 ± 3.05 vs. 2.20 ± 2.70, *p* = 0.033). Males reported significantly higher overall FertiQoL scores than females (54.61 ± 4.79 vs. 52.42 ± 6.39, *p* = 0.019). Increased depression were significantly associated with poorer fertility-related quality of life in males (r = −0.463, *p* < 0.001). Cronbach’s alpha coefficients demonstrated excellent internal consistency for BDI-II (0.905), BAI (0.922), and FertiQoL (0.879). **Conclusions**: Infertile females experience greater anxiety and poorer fertility-related quality of life than males. Psychological distress is strongly associated with reduced fertility-specific quality of life, highlighting the importance of routine psychosocial assessment in infertility care.

## 1. Introduction

Infertility is recognized as a major public health problem affecting millions of couples worldwide and is defined as the inability to achieve a clinical pregnancy after 12 months or more of regular unprotected sexual intercourse [1]. According to the World Health Organization, approximately one in six individuals experiences infertility during their reproductive lifetime, making it a condition with substantial medical, social, and psychological consequences [2]. Beyond its biological implications, infertility is increasingly acknowledged as a life crisis that can profoundly affect emotional well-being, interpersonal relationships, social functioning, and overall quality of life [3].

The experience of infertility is frequently associated with heightened levels of psychological distress. Numerous studies have demonstrated that infertile individuals are at increased risk of anxiety, depression, stress, feelings of inadequacy, reduced self-esteem, and social isolation compared with fertile populations [4,5,6]. The uncertainty surrounding treatment outcomes, repeated treatment failures, financial burden, and societal expectations regarding parenthood may contribute significantly to emotional distress among infertile couples [7]. In some cases, the psychological burden associated with infertility has been reported to be comparable to that observed in patients with serious chronic medical illnesses [8].

Although infertility affects both partners, the psychological impact may not be distributed equally between women and men. Cultural norms and societal expectations often place a greater emphasis on female reproductive capacity, resulting in women experiencing more pronounced emotional consequences following an infertility diagnosis [9]. Several studies have reported higher rates of anxiety and depressive symptoms among infertile women compared with their male partners [10,11]. Women undergoing infertility evaluation and treatment frequently report feelings of guilt, loss of femininity, diminished self-worth, and concerns regarding social stigma [12]. Conversely, infertility may also have substantial psychological consequences for men, particularly in cases of male-factor infertility, where perceptions of masculinity, self-esteem, and sexual identity may be challenged [13,14].

The etiology of infertility may further influence psychological outcomes. Male-factor infertility accounts for approximately 20–30% of infertility cases and contributes to nearly half of all infertility diagnoses when combined with mixed causes [15]. Previous research suggests that psychological responses may vary according to whether infertility is attributed primarily to the male partner, female partner, both partners, or remains unexplained [16]. However, findings remain inconsistent. Some studies indicate that women experience greater psychological distress regardless of infertility etiology, whereas others suggest that men diagnosed with male-factor infertility may exhibit increased depressive symptoms, reduced quality of life, and impaired self-esteem [17,18]. Consequently, the extent to which infertility etiology influences psychological well-being in each partner remains insufficiently understood.

Assessment of quality of life has become an essential component of infertility research. Generic health-related quality-of-life instruments may not adequately capture infertility-specific concerns, leading to the development of specialized tools such as the Fertility Quality of Life (FertiQoL) questionnaire [19]. FertiQoL provides a comprehensive evaluation of emotional, relational, social, and treatment-related dimensions of quality of life among infertile individuals and has been widely validated across different populations [20]. Previous studies have demonstrated significant associations between lower FertiQoL scores and increased anxiety and depressive symptoms, highlighting the close relationship between psychological distress and fertility-related quality of life [21,22].

In addition to infertility etiology, several sociodemographic and clinical characteristics may influence psychological outcomes among infertile couples. Factors such as age, educational level, employment status, duration of infertility, type of infertility, previous treatment experiences, and the presence of living children have all been associated with varying levels of emotional distress and quality of life [23,24,25]. Nevertheless, the relative contributions of these factors remain unclear, and evidence from different populations has yielded heterogeneous results.

Despite growing awareness of the psychosocial dimensions of infertility, limited research has directly compared anxiety, depression, and fertility-specific quality of life between women and men within infertile couples while simultaneously examining the influence of infertility etiology and relevant sociodemographic characteristics. A better understanding of these differences may facilitate the development of targeted psychosocial interventions and individualized support strategies during infertility treatment.

Therefore, the primary aim of the present study was to determine whether anxiety, depression, and quality-of-life scores are higher in women or men among infertile couples and to investigate how these outcomes are affected by factors such as male-factor versus female-factor infertility, presence of living children, educational status, employment status, and age. Secondary objectives included comparing FertiQoL total and subscale scores between women and men, evaluating the relationship between anxiety, depression, and quality of life, and examining the potential effects of sociodemographic and clinical variables on psychological outcomes in infertile couples.

## 2. Materials and Methods

### 2.1. Study Design and Participants

This cross-sectional study consecutively enrolled participants was conducted at the Reproductive Health and Infertility Outpatient Clinic of Mersin City Training and Research Hospital, Türkiye, between March–July 2026. The study aimed to evaluate anxiety, depression, and fertility-related quality of life among infertile couples and to investigate the effects of infertility etiology and selected sociodemographic characteristics on these psychological outcomes.

Couples presenting to the infertility clinic for diagnostic evaluation and treatment were screened consecutively for eligibility. Infertility was defined as the inability to achieve pregnancy despite at least 12 months of regular unprotected sexual intercourse. Women aged 18–45 years, first admission for infertility and their partners who had been diagnosed with either primary or secondary infertility were considered eligible for inclusion. All participants provided written informed consent before enrolment.

Participants were excluded if either partner had a known psychiatric disorder, including major depressive disorder, anxiety disorders, bipolar disorder, psychotic disorders, or other clinically diagnosed psychiatric conditions. Couples were also excluded if either partner was receiving psychotropic medication or had a chronic systemic disease such as diabetes mellitus, malignancy, chronic renal insufficiency, or chronic liver disease. Incomplete questionnaire responses and withdrawal of consent during the study period were also considered exclusion criteria.

### 2.2. Classification of Infertility Etiology

The etiology of infertility was determined according to routine clinical evaluations performed by specialists in Obstetrics and Gynecology and Urology. Based on clinical, laboratory, and radiological findings, participants were categorized into four groups: male-factor infertility, female-factor infertility, combined-factor infertility, and unexplained infertility.

Male-factor infertility was defined as infertility attributable solely to abnormalities in the male partner, including impaired semen parameters, in the absence of any identifiable female infertility factor. Female-factor infertility was defined as infertility caused exclusively by female reproductive disorders, such as ovulatory dysfunction, tubal pathology, endometriosis, or diminished ovarian reserve, while the male partner exhibited normal fertility evaluation results. Combined-factor infertility referred to the presence of infertility-related abnormalities in both partners. Unexplained infertility was diagnosed when no identifiable cause of infertility could be established after standard evaluation of both partners.

### 2.3. Sample Size Calculation

The required sample size was calculated using G*Power software version 3.1 (Heinrich-Heine University, Düsseldorf, Germany). The calculation was based on detecting a clinically meaningful difference in Beck Depression Inventory-II (BDI-II) and Beck Anxiety Inventory (BAI) scores between male and female participants. Assuming a moderate standardized effect size (*dz* = 0.50), a two-sided α level of 0.05, and 80% power, a minimum of 64 infertile couples was required. To account for possible missing data and incomplete questionnaires, 75 couples (a total of 150 participants) were targeted for recruitment.

### 2.4. Data Collection Procedures

Following informed consent, participants completed a standardized data collection form designed for the study. Sociodemographic variables including age, sex, educational status, and employment status were recorded. Clinical characteristics including infertility duration, infertility type (primary or secondary), infertility etiology, and the presence of living children were also documented.

All questionnaires were administered on the same day in a quiet and private environment within the outpatient clinic. Participants completed the questionnaires independently using a self-report format while a researcher was available to provide clarification when necessary. Completed questionnaires were reviewed for completeness before data entry.

### 2.5. Outcome Measures

Fertility-related quality of life was assessed using the Fertility Quality of Life (FertiQoL) questionnaire. FertiQoL is an internationally validated infertility-specific instrument developed by the European Society of Human Reproduction and Embryology (ESHRE) and the American Society for Reproductive Medicine (ASRM). The Turkish validity and reliability study of the FertiQoL questionnaire was conducted by Karabulut et al., demonstrating satisfactory psychometric properties in Turkish infertile populations [22]. The questionnaire consists of a Core module comprising four subscales (Emotional, Mind/Body, Relational, and Social) and a Treatment module comprising two domains (Environment and Tolerability), together with two single-item measures evaluating general physical health and overall life satisfaction. Items are rated on a 5-point Likert scale, and scores were calculated according to the FertiQoL scoring guidelines described by Boivin et al. [19]. Raw item scores were transformed to a standardized 0–100 scale, with higher scores indicating better fertility-related quality of life. Core FertiQoL scores were calculated as the mean of the four Core domains, Treatment FertiQoL scores as the mean of the two Treatment domains, and the total FertiQoL score as the mean of the Core and Treatment scores.

Anxiety symptoms were evaluated using the Beck Anxiety Inventory (BAI), a 21-item self-report scale widely used for the assessment of anxiety severity. Each item is scored on a four-point Likert scale ranging from 0 to 3, resulting in a total score between 0 and 63. Higher scores reflect greater levels of anxiety. The Turkish validity and reliability study of the BAI was conducted by Ulusoy et al., demonstrating satisfactory psychometric properties in the Turkish population [26].

Depressive symptoms were assessed using the Beck Depression Inventory-II (BDI-II), a validated 21-item self-report questionnaire designed to measure the severity of depressive symptoms. Total scores range from 0 to 63, with higher scores indicating more severe depressive symptomatology. The Turkish validity and reliability study of the Beck Depression Inventory was conducted by Hisli, who demonstrated that the Turkish version is a reliable and valid instrument for assessing depressive symptoms in Turkish populations [27].

### 2.6. Data Management and Confidentiality

To ensure confidentiality, all participants were assigned unique identification codes, and no personal identifying information was entered into the study database. Study data were stored in password-protected electronic files accessible only to the research team. All records were maintained in accordance with institutional regulations and applicable data protection standards.

### 2.7. Ethical Considerations

The study was conducted in accordance with the ethical principles outlined in the Declaration of Helsinki. Approval was obtained from the local Institutional Ethics Committee before commencement of participant recruitment. Participation was entirely voluntary, and written informed consent was obtained from all participants. Participants were informed of their right to withdraw from the study at any time without affecting their medical care. Because the study was observational and questionnaire-based, no physical intervention was performed, and no additional clinical risk beyond routine outpatient evaluation was anticipated.

### 2.8. Statistical Analysis

Statistical analyses were performed using IBM SPSS Statistics software (Version 20.0; IBM Corp., Armonk, NY, USA). Continuous variables were expressed as mean ± standard deviation or median (interquartile range) according to data distribution, whereas categorical variables were presented as frequencies and percentages. The normality of continuous variables was assessed using the Shapiro–Wilk test and visual inspection of histograms.

Comparisons between male and female participants were performed using the Wilcoxon signed ranked test with Cohen’s d for effect sizes. Associations between anxiety, depression, and quality-of-life scores were evaluated using Pearson or Spearman correlation analyses according to data distribution where Bonferroni correction was applied for multiple comparisons as *p* < 0.0011. Correlation coefficients were interpreted according to their absolute values as follows: 0.00–0.19, very weak; 0.20–0.39, weak; 0.40–0.59, moderate; 0.60–0.79, strong; and 0.80–1.00, very strong correlation, irrespective of direction. A two-sided *p* value < 0.05 was considered statistically significant.

## 3. Results

Based on the sample size calculation, a minimum of 75 participants was required in each study group. During the study period, 75 infertile couples (150 individuals) who met the eligibility criteria were prospectively and consecutively recruited. A total of 165 participants completed the study questionnaires, and 15 participants were excluded after enrollment because of incomplete data or withdrawal. Consequently, data from all 75 couples (75 females and 75 males) were included in the final analyses.

### 3.1. Reliability Analysis of the Study Instruments

The internal consistency of the psychometric instruments used in the present study was evaluated using Cronbach’s alpha coefficients. Excellent reliability was observed for all scales. Cronbach’s alpha coefficients were 0.905 for the BDI-II, 0.922 for the Beck Anxiety Inventory (BAI), and 0.879 for the Fertility Quality of Life (FertiQoL) questionnaire, indicating high internal consistency of the instruments in the study population.

### 3.2. Demographic and Clinical Characteristics of the Study Population

The mean age was 31.7 ± 5.3 years for females and 34.8 ± 5.9 years for males. The mean duration of marriage was 6.3 ± 4.2 years, and the mean duration of infertility was 3.4 ± 2.5 years. Primary infertility was present in 53 couples (70.7%), whereas 22 couples (29.3%) had secondary infertility. Living children were reported by 20 couples (26.7%), while 55 couples (73.3%) had no children (Table 1).

Regarding infertility etiology, female-factor infertility was the most common diagnosis, affecting 30 couples (40.0%), followed by male-factor infertility in 19 couples (25.3%), combined-factor infertility in 14 couples (18.7%), and unexplained infertility in 12 couples (16.0%) (Table 2).

Among female participants, 45 (60.0%) had completed secondary school or lower education, whereas 30 (40.0%) had a university degree. In contrast, 38 males (50.7%) had secondary school or lower education and 37 (49.3%) had completed university education. Employment rates differed markedly between genders, with 24 females (32.0%) being employed compared with 71 males (94.7%).

Monthly household income was reported as below the minimum wage in 18 couples (24.0%), between the minimum wage and twice the minimum wage in 39 couples (52.0%), and more than twice the minimum wage in 18 couples (24.0%).

### 3.3. Comparison of Anxiety, Depression, and Fertility-Related Quality of Life Between Males and Females

The comparisons of BDI-II, BAI, and FertiQoL scores between male and female participants are presented in Table 3. Females exhibited significantly higher affective depressive symptom scores than males (3.21 ± 3.05 vs. 2.20 ± 2.70, *p* = 0.048), whereas cognitive, somatic, and total BDI-II scores did not differ significantly between genders.

Regarding anxiety, females demonstrated significantly higher neurophysiological (3.84 ± 3.78 vs. 2.48 ± 2.79, *p* = 0.018), subjective (2.58 ± 3.46 vs. 1.33 ± 2.05, *p* = 0.021), autonomic (2.82 ± 3.01 vs. 1.37 ± 2.27, *p* = 0.001), and total BAI scores (10.44 ± 10.04 vs. 6.18 ± 7.86, *p* = 0.005). No significant difference was observed for the panic subscale.

Significant gender differences were also identified in fertility-related quality of life (Table 3). Females reported higher scores in the Emotional, Mind/Body, Social, Tolerability, and Core FertiQoL subscales, whereas males demonstrated significantly higher Relational, Environment, General Physical Health, General Life Satisfaction, Treatment FertiQoL, and overall FertiQoL scores. Overall, males had significantly higher total FertiQoL scores than females (54.61 ± 4.79 vs. 52.42 ± 6.39, *p* = 0.026).

### 3.4. Correlation Between Depressive Symptoms and Fertility-Related Quality of Life

Correlations between depressive symptoms and fertility-related quality of life are presented separately for male participants in Table 4 and female in Table 5. Associated with multiple comparisons, statistical significance was interpreted using the Bonferroni-adjusted threshold (*p* < 0.0011).

Among males, higher depressive symptom scores were consistently associated with poorer fertility-related quality of life, particularly in the relational, environmental, treatment, and overall FertiQoL subscales. Negative moderate correlations were observed between cognitive factor subscale of BDI-II and environmental subscale of FertiQol (r = −0.480, *p* < 0.001), cognitive factor subscale of BDI-II and treatment subscale of FertiQol (r = −0.412, *p* < 0.001), cognitive factor subscale of BDI-II and total FertiQol (r = −0.474, *p* < 0.001), somatic factor subscale of BDI-II and environmental subscale of FertiQol (r = −0.409, *p* < 0.001), affective factor subscale of BDI-II and relational subscale of FertiQol (r = −0.494, *p* < 0.001), affective factor subscale of BDI-II and environmental subscale of FertiQol (r = −0.578, *p* < 0.001), affective factor subscale of BDI-II and treatment subscale of FertiQol (r = −0.547, *p* < 0.001), and total FertiQoL scores (r = −0.474, *p* < 0.001). Likewise, total BDI-II scores showed significant negative moderate correlations with the relational (r = −0.454, *p* < 0.001), environmental (r = −0.535, *p* < 0.001), treatment subscales (r = −0.475, *p*< 0.001), and overall FertiQoL scores (r = −0.463, *p* < 0.001).

Among females, depressive symptoms demonstrated an even moderate association with fertility-related quality of life. Negative moderate correlations were found between cognitive factor subscale of BDI-II and relational subscale of FertiQol (r = −0.465, *p* < 0.001), cognitive factor subscale of BDI-II and environmental subscale of FertiQol (r = −0.601, *p* < 0.001), cognitive factor subscale of BDI-II and treatment subscale of FertiQol (r = −0.578, *p* < 0.001), somatic factor subscale of BDI-II and environmental subscale of FertiQol (r = −0.451, *p* < 0.001), somatic factor subscale of BDI-II and general physical health subscale of FertiQol (r = −0.409, *p* < 0.001), affective factor subscale of BDI-II and relational subscale of FertiQol (r = −0.538, *p* < 0.001), affective factor subscale of BDI-II and environmental subscale of FertiQol (r = −0.643, *p* < 0.001), and affective factor subscale of BDI-II and treatment subscale of FertiQol (r = −0.624, *p* < 0.001). Similarly, total BDI-II scores displayed significant negative moderate correlations with the relational (r = −0.517, *p* < 0.001), environmental (r = −0.635, *p* < 0.001), general physical health (r = −0.405, *p* < 0.001) and treatment subscales of FertiQol (r = −0.630, *p* < 0.001).

### 3.5. Correlation Between Anxiety Symptoms and Fertility-Related Quality of Life

Correlations between anxiety symptoms and fertility-related quality of life are summarized in Table 6 and Table 7. Statistical significance was evaluated using the Bonferroni-adjusted threshold (*p* < 0.0011).

In males, higher anxiety scores were generally associated with poorer fertility-related quality of life. Neurophysiological subscale of BAI showed significant negative moderate correlation with environmental subscale of FertiQol (r = −0.403, *p* < 0.001). Similarly, subjective subscale of BAI demonstrated significant negative correlation with the relational subscale of FertiQol (r = −0.414, *p* < 0.001). Moderate negative correlation was found between autonomic subscale of BAI and environmental (r = −0.414, *p* < 0.001), and general life satisfaction (r = −0.466, *p* < 0.001) subscales of FertiQol.

Among females, anxiety symptoms also showed predominantly negative relationships with fertility-related quality of life. Neurophysiological subscale of BAI demonstrated significant inverse moderate correlations with the relational subscale of FertiQol (r = −0.467, *p* < 0.001), and total FertiQol (r = −0.472, *p* < 0.001). Moderate negative correlation was found between autonomic subscale of BAI and mind/body subscale of FertiQol (r = −0.463, *p* < 0.001). Likewise, significant moderate inverse correlation was shown between totlal BAI and environmental (r = −0.483, *p* < 0.001), and treatment subscales of FertiQol (r = −0.472, *p* < 0.001).

### 3.6. Predictors of Depression, Anxiety, and Fertility-Related Quality of Life in Females

Backward stepwise linear regression analyses were performed to identify factors independently associated with depression, anxiety, and fertility-related quality of life among females (Table 8).

The regression model evaluating predictors of depressive symptoms demonstrated that the number of live births, duration of marriage, infertility duration, educational status, and income level were not independently associated with BDI-II scores (ß = 0.795, *p* = 0.571; ß = −0.084, *p* = 0.793; ß = 0.934, *p* = 0.072; ß = 0.981, *p* = 0.378; ß = −1.738, *p* = 0.286; respectively).

In contrast, infertility duration emerged as an independent predictor of anxiety severity. Specifically, longer infertility duration was significantly associated with higher BAI scores (β = 1.562, *p* = 0.013), whereas the number of live births, duration of marriage, educational status, and income level were not significantly associated with anxiety severity (ß = −0.084, *p* = 0.96; ß = −0.633, *p* = 0.104; ß = −0.204, *p* = 0.879; ß = 0.64, *p* = 0.743; respectively).

Similarly, none of the investigated variables, including number of live births, duration of marriage, infertility duration, educational status, or income level, significantly predicted FertiQoL scores in female participants (ß = 0.483, *p* = 0.663; ß = 0.282, *p* = 0.268; ß = −0.404, *p* = 0.320; ß = −0.833, *p* = 0.344; ß = 0.324, *p* = 0.801; respectively).

### 3.7. Predictors of Depression, Anxiety, and Fertility-Related Quality of Life in Males

Backward stepwise linear regression analyses were performed to identify factors independently associated with depression, anxiety, and fertility-related quality of life among males (Table 8).

The regression model evaluating predictors of depressive symptoms demonstrated that the number of live births, duration of marriage, infertility duration, educational status, and income level were not independently associated with BDI-II scores (ß = 0.548, *p* = 0.699; ß = −0.092, *p* = 0.770; ß = −0.236, *p* = 0.616; ß = 1.345, *p* = 0.318; ß = 1.611, *p* = 0.386; respectively).

In addition, none of the evaluated variables, such as number of live births, duration of marriage, infertility duration, educational status, or income level, significantly predicted FertiQoL scores in male participants (ß = −0.121, *p* = 0.930; ß = −0.070, *p* = 0.819; ß = −0.290, *p* = 0.527; ß = −0.038, *p* = 0.977; ß = 3.448, *p* = 0.060; respectively).

Likewise, none of the evaluated variables, such as number of live births, duration of marriage, infertility duration, educational status, or income level, significantly predicted FertiQoL scores in female participants (ß = −0.066, *p* = 0.938; ß = −0.132, *p* = 0.489; ß = 0.119, *p* = 0.677; ß = −1.208, *p* = 0.140; ß = 0.538, *p* = 0.632; respectively).

## 4. Discussion

The present study investigated sex-related differences in anxiety, depression, and fertility-related quality of life among infertile couples and explored the associations between psychological distress and fertility-specific quality of life. Several noteworthy findings emerged. First, infertile females exhibited significantly higher anxiety levels than their male partners, particularly in neurophysiological, subjective, and autonomic symptom domains. Second, although overall depressive symptom severity was comparable between genders, females reported significantly greater affective depressive symptoms. Third, females demonstrated significantly lower fertility-related quality of life than males across several FertiQoL domains. Finally, both anxiety and depressive symptoms showed significant correlations with fertility-specific quality of life in both genders, suggesting a close interrelationship between emotional well-being and infertility-related experiences.

Collectively, these findings reinforce the conceptualization of infertility as not merely a reproductive disorder but also a multidimensional psychosocial crisis affecting emotional, interpersonal, and social functioning [4,6]. Infertility has been described as a chronic stressor characterized by persistent uncertainty, repeated losses, diminished control over life goals, and disruption of anticipated family trajectories [3]. Consequently, the emotional consequences of infertility frequently extend beyond reproductive concerns and may influence nearly every aspect of an individual’s life.

One of the principal findings of the present study was that females experienced significantly higher levels of anxiety than males. This observation is highly consistent with previous investigations conducted in both Western and non-Western populations. Grammenou et al. reported that women undergoing infertility treatment exhibited a significantly higher prevalence of anxiety disorders compared with male partners [5]. Similarly, Li et al. demonstrated that infertile females consistently reported greater anxiety and psychological distress than males, regardless of treatment stage [6]. Meta-analytic evidence has further confirmed that female gender constitutes one of the strongest predictors of infertility-related emotional distress [4].

Several mechanisms may explain these gender differences. First, females generally bear the greatest physical burden of infertility evaluation and treatment. Even in cases of male-factor infertility, females typically undergo repeated hormonal assessments, ultrasonographic examinations, ovulation induction protocols, oocyte retrieval procedures, and embryo transfer interventions [6]. The cumulative burden associated with these invasive and physically demanding procedures may substantially increase anticipatory anxiety and emotional exhaustion.

Second, sociocultural expectations surrounding parenthood may disproportionately affect females. In many societies, including those characterized by strong pronatalist traditions, motherhood is frequently regarded as a central component of female identity and social status [7]. Consequently, infertility may be perceived by females as a threat to femininity, self-esteem, marital stability, and social acceptance [8]. Feelings of inadequacy, guilt, self-blame, and fear of stigmatization may therefore contribute significantly to elevated anxiety levels [9].

Third, differences in coping styles may partially account for the observed gender disparities. Females tend to employ emotion-focused coping strategies more frequently than males, whereas males are more likely to adopt problem-focused or avoidance-based coping mechanisms [10]. Although emotion-focused coping may facilitate emotional expression, it has also been associated with increased vulnerability to anxiety and depressive symptoms during prolonged infertility treatment [28].

Interestingly, although total depressive symptom severity did not differ significantly between genders, females demonstrated significantly higher affective depressive symptom scores. This finding suggests that infertility affects both partners emotionally, but emotional manifestations may vary according to gender. Previous studies have similarly reported that females are more likely to experience emotional symptoms such as sadness, hopelessness, guilt, and grief reactions following infertility diagnosis [29]. Zurlo et al. even suggested that infertility-related distress among females may be comparable to that experienced by individuals diagnosed with serious medical conditions such as cancer or cardiovascular disease [10].

The lack of significant gender differences in overall depression scores warrants particular consideration. It is possible that males experience psychological distress differently and may underreport depressive symptoms due to sociocultural norms emphasizing emotional restraint and traditional masculine roles [11,12]. Male infertility, in particular, may challenge perceptions of masculinity, virility, and sexual identity, leading to psychological distress that may not be adequately captured by conventional self-report scales [13]. Sahoo et al. demonstrated that infertile males frequently report lower levels of overt emotional distress despite experiencing considerable infertility-related stress and relationship difficulties [14]. Therefore, the absence of significant differences in total depression scores should not be interpreted as evidence that infertility has limited psychological consequences for men.

The present study also identified significant gender differences in fertility-related quality of life. Males reported significantly higher overall FertiQoL scores than females, suggesting a better overall perception of fertility-related quality of life. However, the pattern differed across specific domains. Females demonstrated significantly higher scores in the Emotional, Mind/Body, Social, and Tolerability subscales, as well as in the Core FertiQoL score, whereas males reported higher scores in the Relational, Environment, General Physical Health, General Life Satisfaction, and Treatment FertiQoL domains. These findings indicate that the impact of infertility on quality of life may differ between males and females across specific dimensions rather than uniformly affecting one gender more adversely than the other.

These findings corroborate those of previous studies employing the FertiQoL instrument. Aarts et al. found that infertile females consistently reported lower fertility-related quality-of-life scores than males, particularly within emotional and social domains [21]. Similarly, Karabulut et al. observed marked impairments in emotional functioning, social relationships, and treatment satisfaction among infertile females [22]. Assaysh-Öberg et al. further demonstrated that female gender was independently associated with reduced fertility-related quality of life across diverse cultural settings [23].

An interesting finding of the present study is that females demonstrated significantly higher scores in several Core FertiQoL domains, including emotional, mind/body, and social functioning, yet exhibited lower overall FertiQoL scores than males. This apparent discrepancy can largely be explained by the influence of the Treatment FertiQoL domain, in which females reported significantly lower scores. The overall FertiQoL score incorporates both the Core and Treatment domains; therefore, greater treatment-related burden among females may offset their relatively favorable scores in other aspects of fertility-related quality of life. Infertility treatment is often more physically and emotionally demanding for females, who are typically exposed to repeated hormonal interventions, invasive diagnostic and therapeutic procedures, frequent clinic visits, and greater uncertainty regarding treatment outcomes. Moreover, societal expectations and traditional gender roles may place a disproportionate responsibility for reproductive success on females, further increasing treatment-related stress and perceived burden. Consequently, although females may develop adaptive coping strategies that preserve certain aspects of their emotional and social functioning, the cumulative burden associated with infertility treatment appears to have a substantial negative effect on their overall quality of life. These findings underscore the importance of evaluating specific FertiQoL domains separately rather than relying solely on the total score, as domain-specific analyses may provide a more nuanced understanding of gender differences in the experience of infertility.

Another notable finding was the strong association between psychological distress and fertility-related quality of life. Higher depressive symptom scores were consistently associated with poorer quality of life in both genders, particularly in relational, environmental, and treatment-related domains. Similarly, greater anxiety severity was associated with substantial deterioration in multiple dimensions of FertiQoL.

These findings are in agreement with the theoretical framework underlying the development of the FertiQoL instrument. Boivin et al. proposed that infertility-related quality of life is inherently multidimensional and strongly influenced by emotional well-being [19]. Previous studies have similarly demonstrated that anxiety and depression are among the most important determinants of reduced fertility-related quality of life [24,25]. For example, Aarts et al. reported that depressive symptoms accounted for a substantial proportion of variability in FertiQoL scores among infertile individuals [21].

The observed associations likely reflect a bidirectional relationship. On one hand, infertility-related emotional distress may impair interpersonal communication, sexual relationships, treatment adherence, and social participation, thereby reducing quality of life [28]. On the other hand, persistent reductions in quality of life may further exacerbate anxiety and depressive symptoms, creating a self-perpetuating cycle of psychological distress [29]. This reciprocal interaction underscores the importance of early identification and management of psychological symptoms during infertility treatment.

An additional important finding of the present study was that infertility duration emerged as the sole independent predictor of anxiety severity among females. Longer infertility duration was associated with increased anxiety symptoms, whereas educational level, income status, duration of marriage, and the presence of living children were not independently associated with psychological outcomes. This observation is supported by previous investigations demonstrating that prolonged infertility is associated with increasing psychological burden [30]. Repeated unsuccessful attempts to conceive, cumulative treatment failures, financial costs, and uncertainty regarding treatment outcomes may progressively intensify emotional distress over time [31]. However, previous studies have reported inconsistent findings concerning the influence of sociodemographic variables such as educational level and socioeconomic status [11]. Such discrepancies may reflect cultural differences, variations in healthcare systems, and heterogeneity in study populations.

The clinical implications of the present findings are substantial. Despite remarkable advances in assisted reproductive technologies, infertility management remains predominantly biomedical in many clinical settings. However, our findings clearly indicate that emotional distress and impaired quality of life are highly prevalent among infertile couples, particularly females. Routine psychological screening using validated instruments such as the BAI, BDI-II, and FertiQoL may facilitate early identification of individuals at increased risk of psychological morbidity [29].

Furthermore, multidisciplinary models integrating reproductive specialists, mental health professionals, fertility counsellors, and specialized infertility nurses may significantly improve patient-centered care [31]. Evidence suggests that psychosocial interventions, including cognitive behavioural therapy, mindfulness-based interventions, supportive counselling, and stress-management programs, may reduce psychological distress and improve quality of life among infertile couples [32,33]. Such interventions may also enhance treatment adherence and patient satisfaction throughout the infertility treatment process.

Future longitudinal studies are warranted to clarify causal relationships between infertility-related distress and quality of life. Prospective investigations following couples across different stages of infertility treatment may provide valuable information regarding temporal changes in psychological well-being. In addition, future multicentre studies involving larger and culturally diverse populations are necessary to improve generalizability and to explore the influence of infertility etiology, treatment modality, and reproductive outcomes on psychological adaptation.

Several limitations should be acknowledged. First, the cross-sectional design of the study precludes causal inference regarding the relationship between psychological distress and fertility-related quality of life. Second, although Bonferroni correction was applied to the correlation analyses, the large number of statistical comparisons performed throughout the study increases the possibility of type I error, and therefore some findings should be interpreted with appropriate caution. Third, the single-center design may limit the generalizability of the findings to other populations and healthcare settings. Fourth, psychological outcomes were assessed using self-report instruments rather than structured psychiatric interviews, which may have introduced reporting bias. Fifth, although participants were recruited as infertile couples, males and females were analyzed primarily as separate individuals rather than as dyads. Consequently, the analyses did not account for the potential interdependence of partners’ psychological responses, and future studies using dyadic analytical approaches, such as the Actor–Partner Interdependence Model, are warranted. Sixth, the relatively modest sample size limited detailed subgroup analyses according to infertility etiology and treatment characteristics. Finally, because sociocultural attitudes toward infertility vary considerably across populations, caution is warranted when generalizing these findings beyond the Turkish context.

## 5. Conclusions

In conclusion, infertile females experienced significantly greater anxiety and poorer fertility-related quality of life than males, whereas overall depressive symptom severity was largely comparable between genders. Anxiety and depressive symptoms were strongly associated with fertility-specific quality of life in both partners, highlighting the multidimensional psychosocial burden of infertility. Comprehensive infertility care should therefore incorporate routine psychological assessment and individualized psychosocial support alongside medical treatment in order to optimize both emotional well-being and treatment experiences.

## Figures and Tables

**Table 1:** Demographics.

		Male (Mean ± SD)	Female (Mean ± SD)	*p*
**Age**		32.22 ± 6.21	29.26 ± 4.53	0.001 *
**Infertility duration**		—	3.2 ± 2.4	
**Duration of Marriage**		—	4.85 ± 3.7	
		***n*** **(%)**	***n*** **(%)**	***p***
**Number of pregnancy**	**0**	—	54 (72)	—
	**1**	—	12 (16)	
	**2**	—	6 (8)	
	**3**	—	2 (2.7)	
	**>4**	—	1 (1.3)	
**Number of delivery**	**0**	—	63 (84)	—
	**1**	—	5 (6.7)	
	**2**	—	6 (8)	
	**>3**	—	1 (1.3)	
**Number of miscarriages**	**0**	—	62 (82.7)	—
	**1**	—	11 (14.7)	
	**2**	—	1 (1.3)	
	**3**	—	1 (1.3)	
**Number of live births**	**0**	—	60 (80)	—
	**1**	—	10 (13.3)	
	**2**	—	4 (5.3)	
	**>3**	—	1 (1.3)	
**Number of shared children**	**0**	—	61 (81.3)	—
	**1**	—	8 (10.7)	
	**2**	—	5 (6.7)	
	**>3**	—	1 (1.3)	
**Educational status**	**None**	—	1 (1.3)	—
	**Literate**	—	5 (6.7)	
	**Primary**	14 (18.7)	19 (25.3)	
	**High school**	33 (44)	23 (30.7)	
	**University**	28 (37.3)	27 (36)	
**Chronic disease**	**Present**	4 (5.3)	5 (6.7)	0.461
**Income**	**Income < Expense**	1 (1.3)	18 (24)	0.00002 *
	**Income = Expense**	35 (46.7)	42 (56)	
	**Income > Expense**	39 (52)	15 (20)	

* *p* < 0.05 (significant differences indicated in bold). Student *t* test. Chi-square test.

**Table 2:** Infertility characteristics.

		Male *n* (%)	Female *n* (%)	*p*
**Infertility type**	**Primary**	—	54 (72)	—
	**Secondary**	—	21 (28)	—
**Cause of infertility**	**Ovulation problem**	—	19 (25.3)	—
	**Tubal factor**	—	7 (9.3)	—
	**Endometrial factor**	—	4 (5.3)	—
	**Male factor**	—	9 (12)	—
	**Unexplained infertility**	—	32 (42.7)	—
	**Both of male and female factor**	—	4 (5.3)	—
**Previous history of IVF treatment**	**Present**	—	45 (60)	—

**Table 3:** The comparison of BAI, BDI-II and FertiQoL questionnaires.

		Male (Mean ± SD)	Female (Mean ± SD)	Cohen’s d	95% CI	*p*
**BDI-II** **(0–63)**	**Cognitive factor**	2.16 ± 3.57	2.64 ± 3.74	0.088	−1.73–0.77	0.448
	**Somatic factor**	1.85 ± 2.35	2.29 ± 2.36	0.117	−1.29–0.41	0.306
	**Affective factor**	2.2 ± 2.7	3.21 ± 3.05	0.22	−2.04–0.02	0.048 *
	**BDI-II-Total**	6.21 ± 7.92	8.14 ± 8.31	0.15	−4.86–0.99	0.144
**BAI** **(0–63)**	**Neurophysiological subscale**	2.48 ± 2.79	3.84 ± 3.78	0.26	−2.52–−0.19	0.018 *
	**Subjective subscale**	1.33 ± 2.05	2.58 ± 3.46	0.29	−2.22–−0.28	0.021 *
	**Autonomic subscale**	1.37 ± 2.27	2.82 ± 3.01	0.4	−2.28–−0.62	0.001 *
	**Panic subscale**	1 ± 1.91	1.18 ± 1.65	0.06	−0.81–0.43	0.216
	**BAI-Total**	6.18 ± 7.86	10.44 ± 10.04	0.31	−7.33–−1.17	0.005 *
**FertiQol** **(0–100)**	**Emotional subscale**	57 ± 8.58	62.77 ± 6.49	0.55	−8.17–−3.38	0.00002 *
	**Mind/Body subscale**	43.55 ± 9.59	54.94 ± 9.82	0.83	−14.5–−8.27	<0.001 *
	**Relational subscale**	62.77 ± 15.98	51.66 ± 18.92	0.4	4.85–17.36	0.001 *
	**Social subscale**	40.05 ± 10.49	47.08 ± 12.4	0.41	−10.93–−3.13	0.001 *
	**Environment subscale**	71.44 ± 18.14	62.33 ± 18.46	0.32	2.6–15.61	0.009 *
	**Tolerability subscale**	52 ± 6.9	54.42 ± 5.35	0.28	−4.37–−0.47	0.025 *
	**General physical health**	79.33 ± 19.44	69.66 ± 14.41	0.38	3.92–15.41	0.001 *
	**General life satisfaction**	73 ± 20.03	66 ± 17.74	0.25	0.61–13.38	0.047 *
	**Core FertiQol-Total**	50.84 ± 4.44	52.8 ± 5.75	0.25	−3.69–−0.22	0.031 *
	**Treatment FertiQol-Total**	63.66 ± 10.81	58.33 ± 11.46	0.31	1.4–9.26	0.011 *
	**FertiQol-Total**	54.61 ± 4.79	52.42 ± 6.39	0.25	0.24–4.14	0.026 *

* *p* < 0.05 (significant differences indicated in bold). Wilcoxon signed rank test. BDI-II, Beck depression inventory; BAI, Beck anxiety inventory; FertiQol, fertility and quality of life; CI, confidence interval.

**Table 4:** Correlations between BDI-II and FertiQol measurements in males.

	EM (r, *p*)	MB (r, *p*)	RE (r, *p*)	SO (r, *p*)	EN (r, *p*)	TO (r, *p*)	GPH (r, *p*)	GLS (r, *p*)	Core (r, *p*)	Tr (r, *p*)	Total (r, *p*)
**CF-BDI-II**	−0.196 0.091	−0.109 0.351	**−0.383** <0.001 *	−0.175 0.134	**−0.480** <0.001 *	−0.281 0.015	−0.068 0.560	−0.264 0.022	−0.277 0.016	**−0.412** <0.001 *	**−0.454** **<** 0.001 *
**SF-BDI-II**	−0.169 0.148	−0.282 0.014	**−0.379** 0.0007 *	−0.207 0.075	**−0.409** <0.001 *	−0.263 0.023	−0.045 0.701	−0.178 0.126	−0.148 0.205	−0.345 0.002	−0.326 0.004
**AF-BDI-II**	−0.226 0.051	−0.363 0.0013	**0.494** <0.001 *	−0.317 0.006	**−0.578** <0.001 *	−0.137 0.242	−0.004 0.974	−0.199 0.088	−0.170 0.145	**−0.547** <0.001 *	**−0.474** **<** 0.001 *
**T-BDI-II**	−0.216 0.063	−0.257 0.026	**−0.454** <0.001 *	−0.248 0.032	**−0.535** <0.001 *	−0.251 0.030	−0.046 0.698	−0.240 0.038	−0.227 0.05	**−0.475** <0.001 *	**−0.463** **<** 0.001 *

* *p* < 0.0011 (after Bonferroni correction, significant differences indicated in bold). Pearson correlation analysis (r). BDI-II, Beck depression inventory-II; CF-BDI-II, cognitive factor; SF-BDI-II, somatic factor; AF-BDI,-II affective factor; T-BDI-II, total; FTQ, fertility quality of life; EM, emotional subscale; MB, mind/body subscale; RE, relational subscale; SO, social subscale; EN, environmental subscale; TO, tolerability subscale; GPH, general physical health subscale; GLS, general life satisfaction; Core, core; Tr, treatment.

**Table 5:** Correlations between BDI-II and FertiQol measurements in females.

	EM (r, *p*)	MB (r, *p*)	RE (r, *p*)	SO (r, *p*)	EN (r, *p*)	TO (r, *p*)	GPH (r, *p*)	GLS (r, *p*)	Core (r, *p*)	Tr (r, *p*)	Total (r, *p*)
**CF-BDI-II**	−0.025 0.832	−0.099 0.396	**−0.465** <0.001 *	−0.361 0.0014	**−0.601** <0.001 *	−0.034 0.769	−0.349 0.002	−0.308 0.007	−0.102 0.383	**−0.578** <0.001 *	−0.325 0.004
**SF-BDI-II**	−0.170 0.144	−0.254 0.028	**−0.385** 0.0006 *	−0.149 0.202	**−0.451** <0.001 *	−0.136 0.243 *	**−0.409** <0.001 *	**−0.394** <0.001 *	−0.147 0.208	−0.494 0.002	−0.249 0.031
**AF-BDI-II**	−0.160 0.171	−0.212 0.067	**0.538** <0.001 *	**−0.381** 0.0007 *	**−0.643** <0.001 *	−0.099 0.397	−0.358 0.002	**−0.388** 0.0005 *	−0.08 0.493	**−0.624** <0.001 *	−0.321 0.005
**T-BDI-II**	−0.096 0.413	−0.195 0.093	**−0.517** <0.001 *	−0.345 0.002	**−0.635** <0.001 *	−0.091 0.438	**−0.405** <0.001 *	**−0.394** <0.001 *	−0.117 0.315	**−0.630** <0.001 *	−0.335 0.003

* *p* < 0.0011 (after Bonferroni correction, significant differences indicated in bold). Pearson correlation analysis (r). BDI-II, Beck depression inventory; CF-BDI-II, cognitive factor; SF-BDI-II, somatic factor; AF-BDI-<<ıı, affective factor; T-BDI-II, total; FTQ, fertility quality of life; EM, emotional subscale; MB, mind/body subscale; RE, relational subscale; SO, social subscale; EN, environmental subscale; TO, tolerability subscale; GPH, general physical health subscale; GLS, general life satisfaction; Core, core; Tr, treatment.

**Table 6:** Correlations between BAI and FertiQol measurements in males.

	EM (r, *p*)	MB (r, *p*)	RE (r, *p*)	SO (r, *p*)	EN (r, *p*)	TO (r, *p*)	GPH (r, *p*)	GLS (r, *p*)	Core (r, *p*)	Tr (r, *p*)	Total (r, *p*)
**NF-BAI**	−0.100 0.395	−0.256 0.041	**−0.390** 0.0005 *	−0.286 0.013	**−0.403** <0.001 *	−0.059 0.615	−0.039 0.741	**−0.393** <0.001 *	−0.102 0.383	**−0.391** 0.0005 *	−0.326 0.004
**SS-BAI**	−0.102 0.384	−0.247 0.032	**−0.406** <0.001 *	−0.171 0.142	−0.324 0.005	−0.042 0.723	−0.070 0.548	−0.180 0.121	−0.179 0.124	−0.316 0.006	−0.326 0.004
**AS-BAI**	−0.182 0.118	−0.293 0.011	−0.349 0.002	−0.205 0.078	**−0.414** <0.001 *	−0.092 0.433	−0.175 0.133	**−0.466** <0.001 *	−0.122 0.296	**−0.394** <0.001 *	−0.341 0.003
**T-BAI**	−0.137 0.240	−0.283 0.014	−0.315 0.006	−0.213 0.066	−0.354 0.002	−0.307 0.007	−0.091 0.438	−0.221 0.057	−0.071 0.544	−0.278 0.016	−0.231 0.046

* *p* < 0.0011 (after Bonferroni correction, significant differences indicated in bold). Pearson correlation analysis (r). BAI, Beck anxiety inventory; NF-BAI, neurophysiological subscale; SS-BAI, subjective subscale; AS-BAI, autonomic subscale; T-BAI, total; FTQ, fertility quality of life; EM, emotional subscale; MB, mind/body subscale; RE, relational subscale; SO, social subscale; EN, environmental subscale; TO, tolerability subscale; GPH, general physical health subscale; GLS, general life satisfaction; Core, core; Tr, treatment.

**Table 7:** Correlations between BAI and FertiQol measurements in females.

	EM (r, *p*)	MB (r, *p*)	RE (r, *p*)	SO (r, *p*)	EN (r, *p*)	TO (r, *p*)	GPH (r, *p*)	GLS (r, *p*)	Core (r, *p*)	Tr (r, *p*)	Total (r, *p*)
**NF-BAI**	−0.081 0.492	−0.353 0.002	**−0.467** <0.001 *	−0.288 0.012	−0.360 0.002	−0.200 0.086	**−0.443** <0.001 *	−0.298 0.009	−0.05 0.671	**−0.411** <0.001 *	−0.306 0.008
**SS-BAI**	−0.113 0.335	−0.276 0.016	**−0.559** <0.001 *	−0.276 0.016	−0.355 0.002	−0.195 0.093	**−0.370** 0.0010 *	−0.149 0.201	−0.154 0.188	**−0.399** <0.001 *	**−0.472** <0.001 *
**AS-BAI**	−0.126 0.280	**−0.463** <0.001 *	−0.307 0.007	−0.189 0.104	−0.123 0.293	−0.283 0.014	−0.356 0.002	−0.213 0.067	−0.085 0.466	−0.212 0.068	−0.163 0.162
**T-BAI**	−0.102 0.386	−0.088 0.454	−0.295 0.01	−0.095 0.416	**−0.483** <0.001 *	−0.034 0.771	−0.241 0.038	−0.287 0.013	−0.22 0.058	**−0.472** <0.001 *	−0.290 0.012

* *p* < 0.0011 (after Bonferroni correction, significant differences indicated in bold). Pearson correlation analysis (r). BAI, Beck anxiety inventory; NF-BAI, neurophysiological subscale; SS-BAI, subjective subscale; AS-BAI, autonomic subscale; T-BAI, total; FTQ, fertility quality of life; EM, emotional subscale; MB, mind/body subscale; RE, relational subscale; SO, social subscale; EN, environmental subscale; TO, tolerability subscale; GPH, general physical health subscale; GLS, general life satisfaction; Core, core; Tr, treatment.

**Table 8:** Regression analysis for possible predictors on BDI-II, BAI and FertiQol scores of males and females.

Males		Beta	95% CI	*p*
**BDI-II**	**Number of live births**	0.548	−2.26–3.35	0.699
	**Duration of marriage**	−0.092	−0.71–0.53	0.77
	**Infertility duration**	−0.236	−1.17–0.69	0.616
	**Educational status**	1.345	−1.32–4.01	0.318
	**Income level**	1.611	−2.076–5.298	0.386
**BAI**	**Number of live births**	0.795	−1.99–3.58	0.571
	**Duration of marriage**	−0.084	−0.72–0.55	0.793
	**Infertility duration**	0.934	−0.08–1.95	0.072
	**Educational status**	0.981	−1.22–3.18	0.378
	**Income level**	−1.738	−4.96–1.48	0.286
**FertiQol**	**Number of live births**	−0.066	−0.07–0.93	0.938
	**Duration of marriage**	−0.132	−0.69–0.48	0.489
	**Infertility duration**	0.119	0.41–0.67	0.677
	**Educational status**	−1.208	−1.49–0.14	0.14
	**Income level**	0.538	0.48–0.63	0.481
**Females**		**Beta**	**95% CI**	***p***
**BDI-II**	**Number of live births**	0.795	−1.99–3.58	0.571
	**Duration of marriage**	−0.084	−0.72–0.55	0.793
	**Infertility duration**	0.934	−0.08–1.95	0.072
	**Educational status**	0.981	−1.22–3.18	0.378
	**Income level**	−1.738	−4.96–1.48	0.286
**BAI**	**Number of live births**	−0.084	−3.43–3.27	0.960
	**Duration of marriage**	−0.633	−1.4–0.13	0.104
	**Infertility duration**	1.562	0.33–2.78	0.013 *
	**Educational status**	−0.204	−2.85–2.44	0.879
	**Income level**	0.64	−3.23–4.51	0.743
**FertiQol**	**Number of live births**	0.483	−1.72–2.68	0.663
	**Duration of marriage**	0.282	−0.22–0.78	0.268
	**Infertility duration**	−0.404	−1.2–0.4	0.320
	**Educational status**	0.833	−2.57–0.91	0.344
	**Income level**	0.324	−2.22–2.87	0.801

* *p* < 0.05 (significant differences indicated in bold). Linear regression model. BDI-II, Beck depression inventory-II; BAI, Beck anxiety inventory; FertiQol, fertility and quality of life.

## Data Availability

It is prohibited to transfer, rent or sell the research data to any third party other than the researcher who has been officially approved for database use.

## Abbreviations

The following abbreviations are used in this manuscript:

BAI	Beck Anxiety Inventory
BDI-II	Beck Depression Inventory-II
FertiQoL	Fertility Quality of Life
ESHRE	European Society of Human Reproduction and Embryology
ASRM	American Society for Reproductive Medicine
